# A Rare Case of an Isolated Interventricular Septal Hydatid Cyst Presenting With Atrioventricular Block in a Non-endemic Region

**DOI:** 10.7759/cureus.102673

**Published:** 2026-01-30

**Authors:** Mehran Jalilzadehbinazar, Maged Y Haikal, Matthew L German, Jeremy E Leidenfrost, Anupama K Rao

**Affiliations:** 1 Cardiology, St Luke's Hospital, Chesterfield, USA; 2 Infectious Diseases, St Luke's Hospital, Chesterfield, USA; 3 Cardiac Surgery, St Luke's Hospital, Chesterfield, USA

**Keywords:** atrioventricular block, cardiac echinococcosis, hydatid cyst, intra-cardiac mass, non-endemic region

## Abstract

Cardiac involvement in human echinococcosis is rare, with isolated interventricular septal (IVS) cysts representing an uncommon presentation. This report details the case of a 41-year-old male who presented to urgent care with fever, cough, and exertional dyspnea. Initial evaluation revealed high-grade atrioventricular (AV) block and markedly elevated inflammatory markers. Advanced cardiac imaging (echocardiography, CT, and MRI) identified a large, complex cystic mass within the basal IVS. Serology was positive for *Echinococcus* antibody, and a history of residence in an endemic region with contact with stray dogs provided epidemiological support. The patient underwent successful surgical excision of the cyst followed by adjuvant albendazole therapy, with an uncomplicated recovery. This case highlights the diagnostic challenge of cardiac hydatid disease, which can mimic sepsis or myocarditis, and emphasizes the critical role of multimodal imaging and exposure history in guiding diagnosis. Given the risk of life-threatening complications, timely surgical intervention combined with medical therapy is essential for management.

## Introduction

Human echinococcosis, primarily caused by *Echinococcus granulosus*, is a zoonotic parasitic infection endemic to pastoral regions worldwide, including the Mediterranean, South America, Eastern Europe, the Middle East, Africa, and parts of Asia [1,2]. While the liver and lungs are the most commonly affected organs, cardiac involvement is rare, with a reported prevalence ranging from 0.02% to 2% of all hydatid disease cases [2,3]. Among cardiac locations, the left ventricular free wall is most frequently involved. Isolated involvement of the interventricular septum (IVS) is particularly uncommon, accounting for approximately 4% of cardiac hydatid cysts [4,5].

The clinical presentation of cardiac echinococcosis is highly variable and often nonspecific, posing significant diagnostic challenges. Symptoms such as chest pain, dyspnea, palpitations, and fever can mimic other cardiac pathologies, including tumors, abscesses, or ischemic events [2,6,7]. In many instances, patients remain asymptomatic until the development of serious complications [4]. The diagnostic workup is further complicated by the limitations of serological testing, which can yield false-negative results, particularly in cases of intact, isolated, or calcified cysts [8,9].

Complications arising from cardiac hydatid cysts can be catastrophic and life-threatening. Cyst rupture may lead to anaphylactic shock, pericardial tamponade, or systemic or pulmonary embolism [2,3,6,10]. Due to their location, cysts involving the interventricular septum are notably associated with conduction disturbances and arrhythmias, including high-grade atrioventricular (AV) block, which may be the initial presenting symptom [8,11,12]. Other potential complications include outflow tract obstruction, valvular dysfunction, and congestive heart failure [2,13].

Given the potential for severe morbidity and mortality, a high index of suspicion is required for timely diagnosis, especially in non-endemic regions or in patients with remote exposure histories. We report a challenging case of a large, isolated hydatid cyst within the IVS, presenting as a febrile illness complicated by high-grade AV block, and detail the diagnostic pathway and successful multidisciplinary management.

## Case presentation

A 41-year-old, previously healthy and physically active male presented to urgent care with a persistent fever and dry cough of several days’ duration. He reported new-onset exertional dyspnea during his regular runs, beginning one week prior. He denied recent domestic or international travel, tick bites, or insect exposures. Although he had experienced occasional left flank pain in the past, it was absent at presentation. At urgent care, he was found to have an irregular heart rhythm and persistent fever, prompting referral to our emergency department.

**Figure 1:**
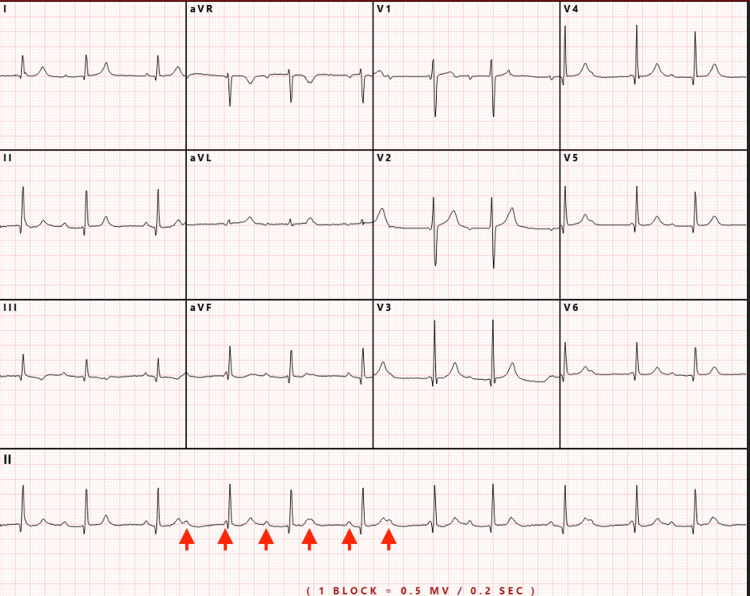
EKG with AV block and junctional escape rhythm EKG: electrocardiogram; AV: atrioventricular

Upon arrival, he was febrile (40.1 °C) and hypotensive (86/58). After initial fluid resuscitation with crystalloids, his blood pressure stabilized. Physical examination revealed a regularly irregular rhythm. An electrocardiogram (ECG) showed high-grade AV dissociation with a junctional escape rhythm (Figure 1). Laboratory findings included a white blood cell count of 6.0 × 10³/μL, hemoglobin 11.0 g/dL, hematocrit 33.2%, mean corpuscular volume (MCV) 87.1 fL, and mean corpuscular hemoglobin (MCH) 28.9 pg. Cardiac troponin I was elevated at 2.47 ng/mL. Inflammatory markers were markedly elevated, with CRP at 5.2 mg/dL and procalcitonin at 24.5 ng/mL. Chest X-ray and urinalysis were unremarkable.

**Figure 2:**
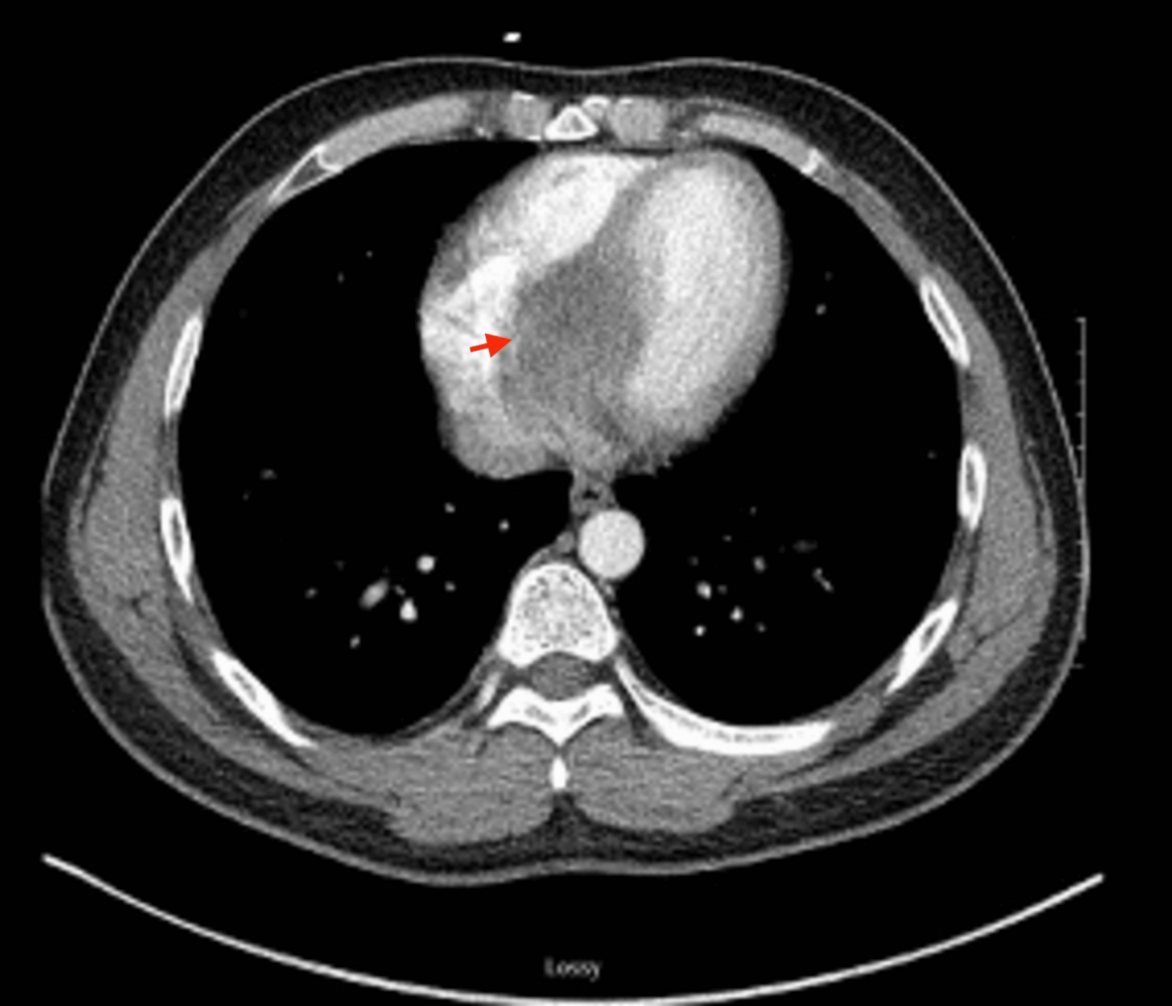
Non-gated chest CT showing a large hypoattenuating mass in the interventricular septum

Given the fever, elevated inflammatory markers, and conduction abnormality, broad-spectrum antibiotics were initiated after obtaining blood cultures, and the patient was admitted. A chest CT scan to evaluate for pulmonary infection unexpectedly revealed an intraventricular septal mass (Figure 2).

Further history revealed that the patient had lived in India from 2013 to 2016. While he denied pet ownership or farm exposure, he reported frequent contact with stray dogs owned by a friend during that period. He had also taken cruises to Jamaica and Haiti (early 2024), and to Honduras and Mexico (2021), but recalled no animal exposures during those trips.

**Figure 3:**
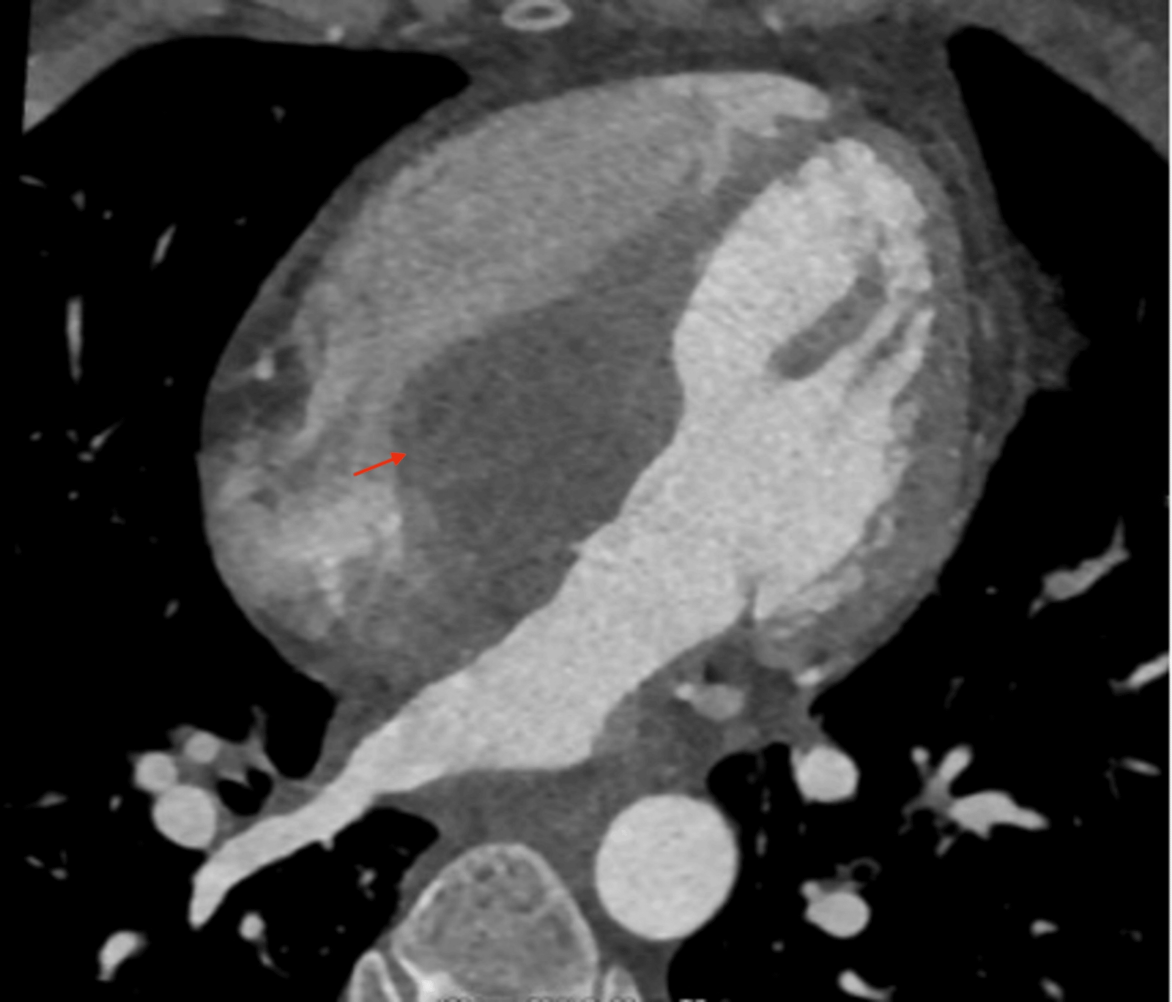
Gated coronary CTA showed a hypoattenuating structure located in the basal interventricular septum, adjacent to the tricuspid valve, with pre-contrast HU of 3 HU and post-contrast HU of 40-60 HU, suggesting a fluid-based mass with possible vascularity CTA: computed tomography angiography

Gated coronary CTA showed a hypoattenuating structure located in the basal interventricular septum, adjacent to the tricuspid valve, with pre-contrast HU 3 HU and post-contrast HU 40-60 HU, suggesting a fluid-based mass with possible vascularity. (Figure 3).

**Figure 4:**
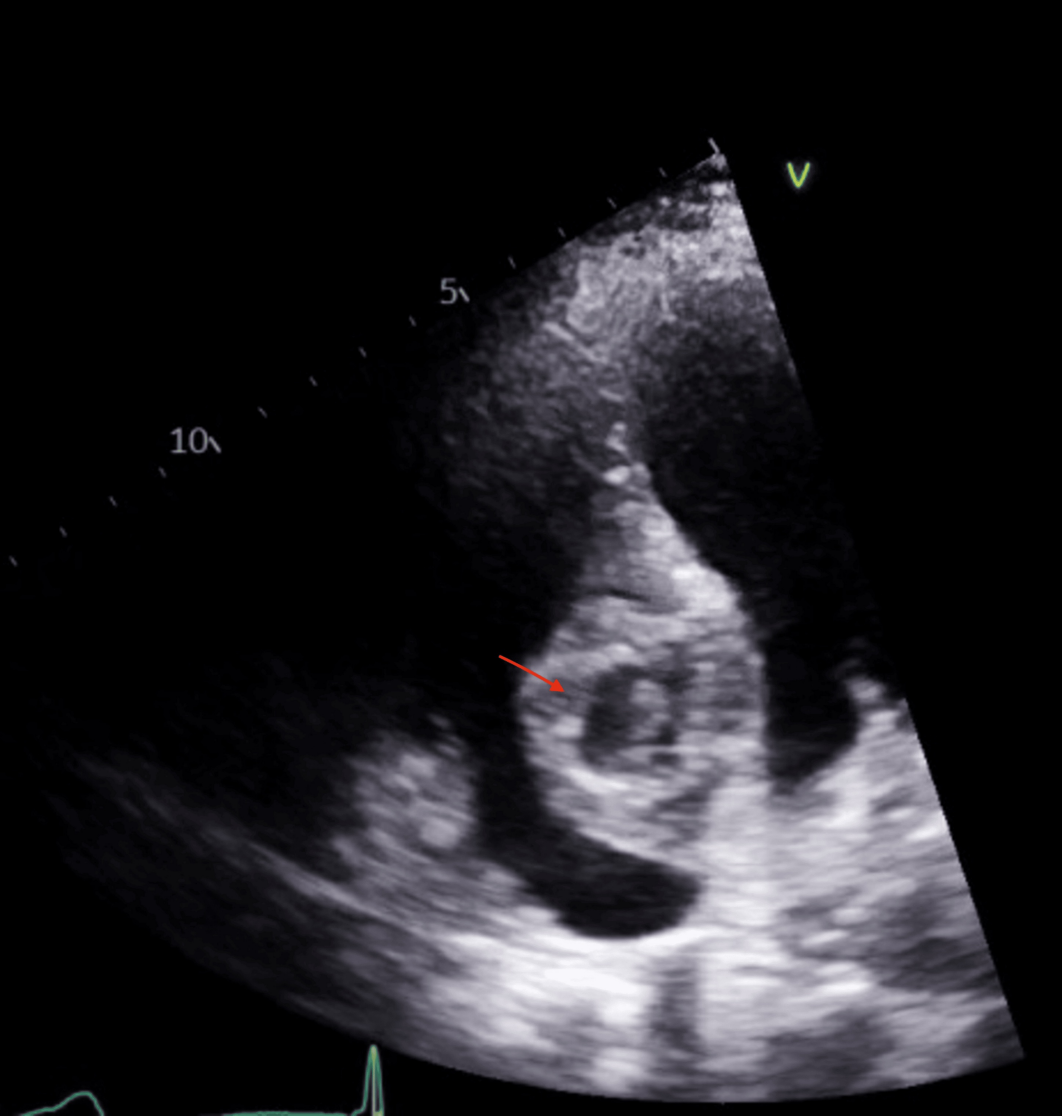
Apical four-chamber view on TTE showing complex cystic mass in the basal interventricular septum, close to the tricuspid annulus/right atrium TTE: transthoracic echocardiography

**Figure 5:**
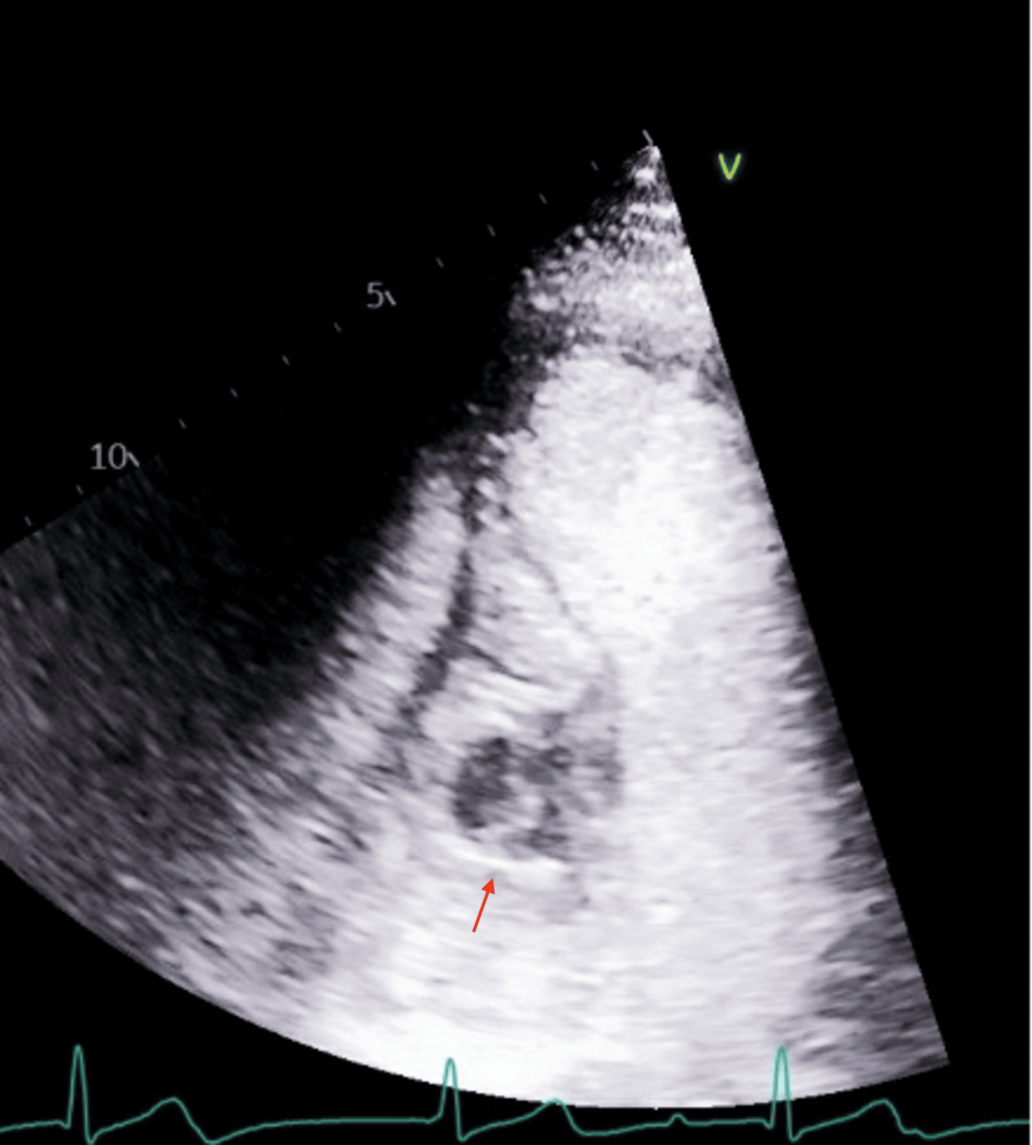
Apical four-chamber view on TTE with ultrasound-enhancing agent (UEA) showing a complex mass with a central region of non-enhancement and heterogeneous contrast uptake, suggesting a cystic component TTE: transthoracic echocardiography

**Figure 6:**
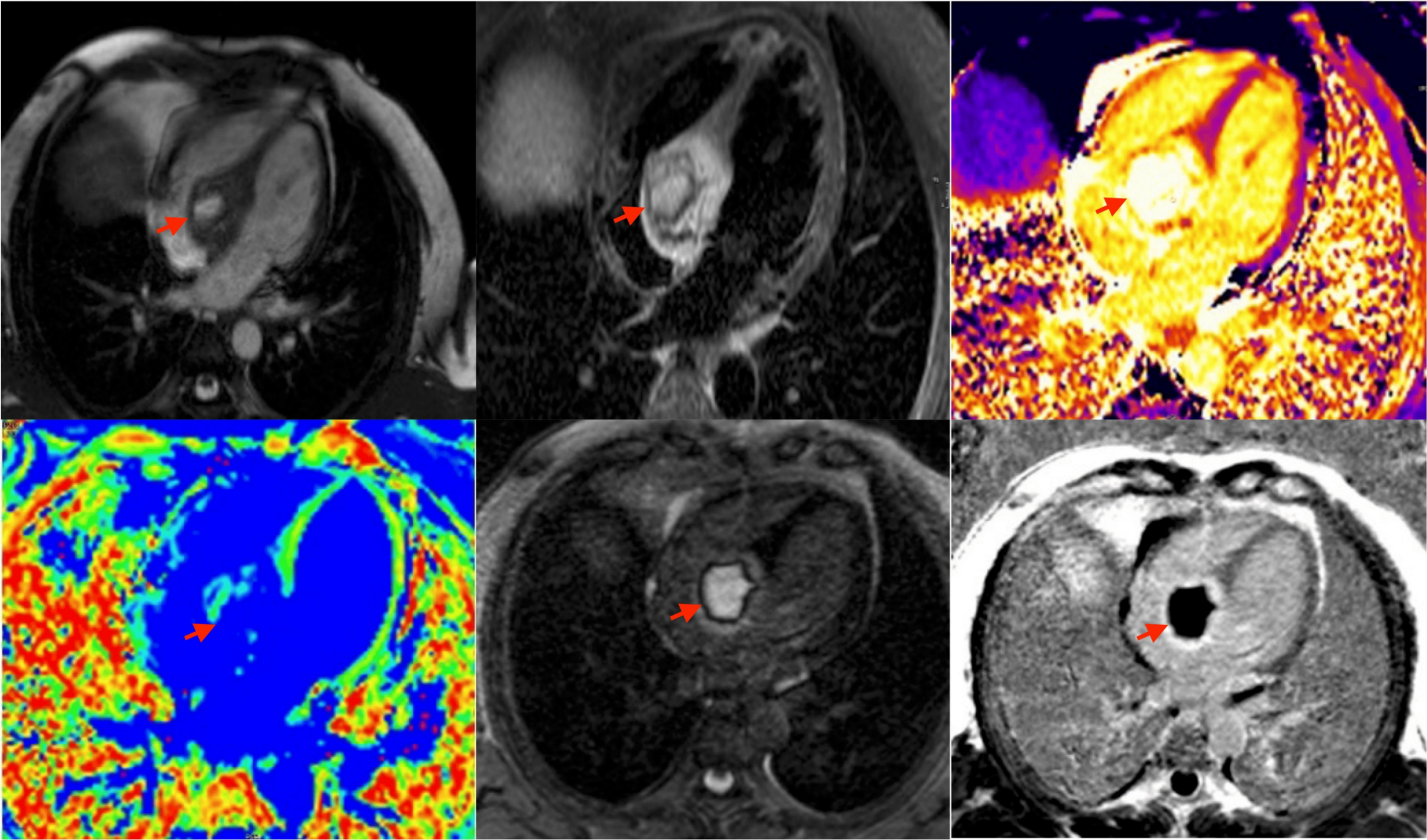
From left to right and top to bottom: SSFP cine image, T2 STIR, T1 map, T2 map, Mag IR LGE, and PSIR LGE CMR showed SSFP cine images with a heterogeneous mass involving the interventricular septum; a T2 STIR image with a hyperintense structure suggesting a mass; T1 mapping demonstrating a central core of the mass with elevated T1 (3,795 ms) and a peripheral region with T1 of 1,992 ms, suggesting a fluid-filled core; and T2 mapping showing a central core with elevated T2 (99 ms) and a peripheral region also with elevated T2 (92 ms), both suggestive of a cystic quality. Mag IR and PSIR late gadolinium-enhancement images confirmed central hypoenhancement. Overall, these tissue characteristics were consistent with a cystic mass. SSFP: steady-state free precession; STIR: short tau inversion recovery; CMR: cardiovascular magnetic resonance; IR: infrared; PSIR: phase-sensitive inversion recovery

Transthoracic echocardiography (TTE) demonstrated a large, complex mass within the basal septum measuring approximately 3.2 × 4.4 cm with an internal cavity. TTE with ultrasound-enhancing agent (UEA) showed a complex mass with a central region of non-enhancement and heterogeneous contrast uptake, suggesting a cystic component (Figures 4, 5). Transesophageal echocardiography (TEE) confirmed a well-circumscribed mass within the basal to mid-IVS, extending into the right atrium and ventricle, without significant valvular impingement. Cardiac MRI with gadolinium revealed a 3.9 × 5.0 cm mass involving the basal to mid-IVS and the inferior anterior atrial septum, protruding into the right atrium. Cardiovascular magnetic resonance (CMR) showed steady-state free precession (SSFP) cine images with a heterogeneous mass involving the interventricular septum; a T2 short tau inversion recovery (STIR) image with a hyperintense structure suggesting a mass; T1 mapping demonstrating a central core of the mass with elevated T1 (3,795 ms) and a peripheral region with T1 of 1,992 ms, suggesting a fluid-filled core; and T2 mapping showing a central core with elevated T2 (99 ms) and a peripheral region also with elevated T2 (92 ms), both suggestive of a cystic quality. Mag infrared (IR) and phase-sensitive inversion recovery (PSIR) late gadolinium enhancement images confirmed central hypoenhancement. Overall, these tissue characteristics were consistent with a cystic mass (Figure 6). Differential diagnoses included cystic neoplasm, myocardial abscess, and parasitic cyst.

**Figure 7:**
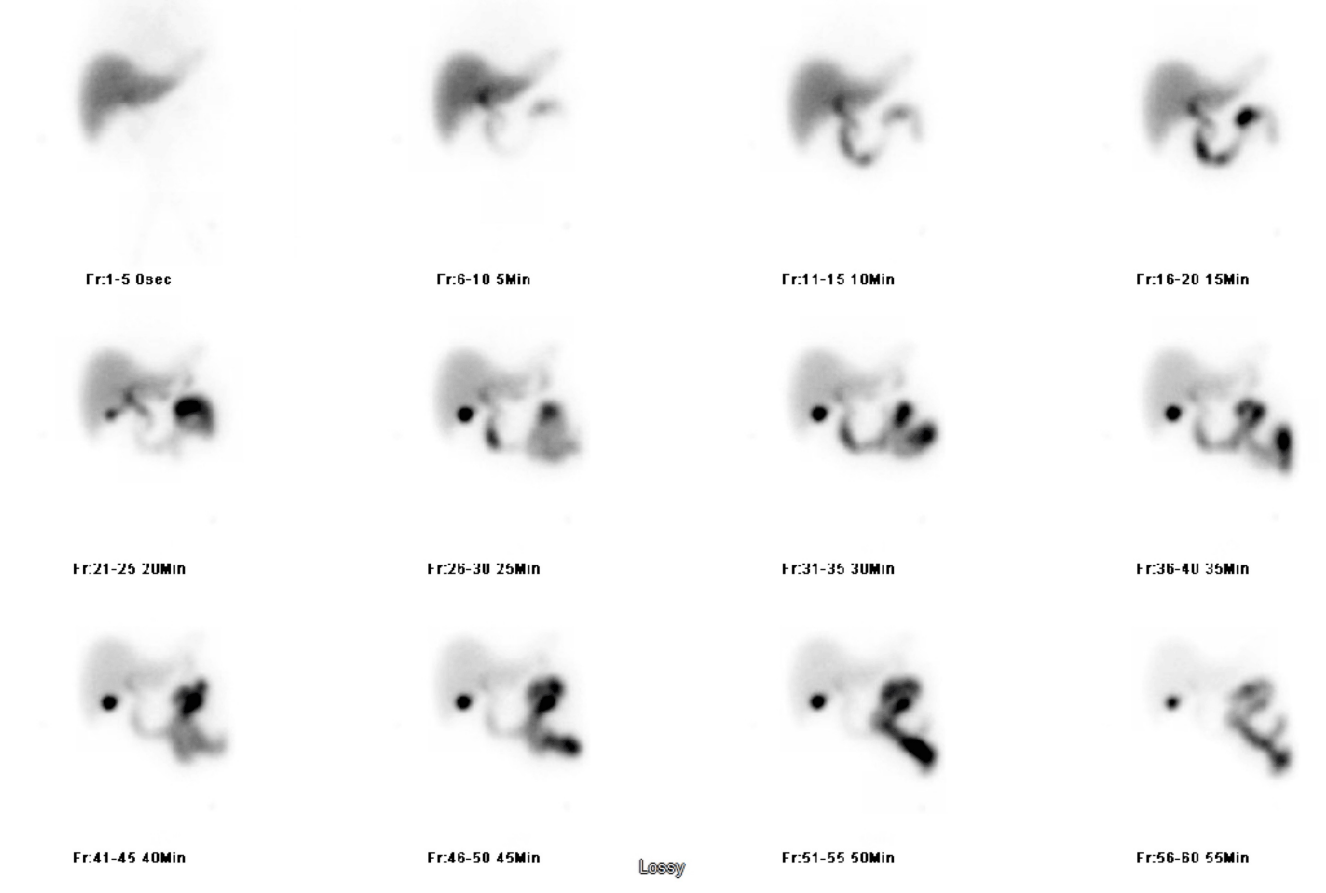
Hepatobiliary scan showing no hepatic involvement

**Figure 8:**
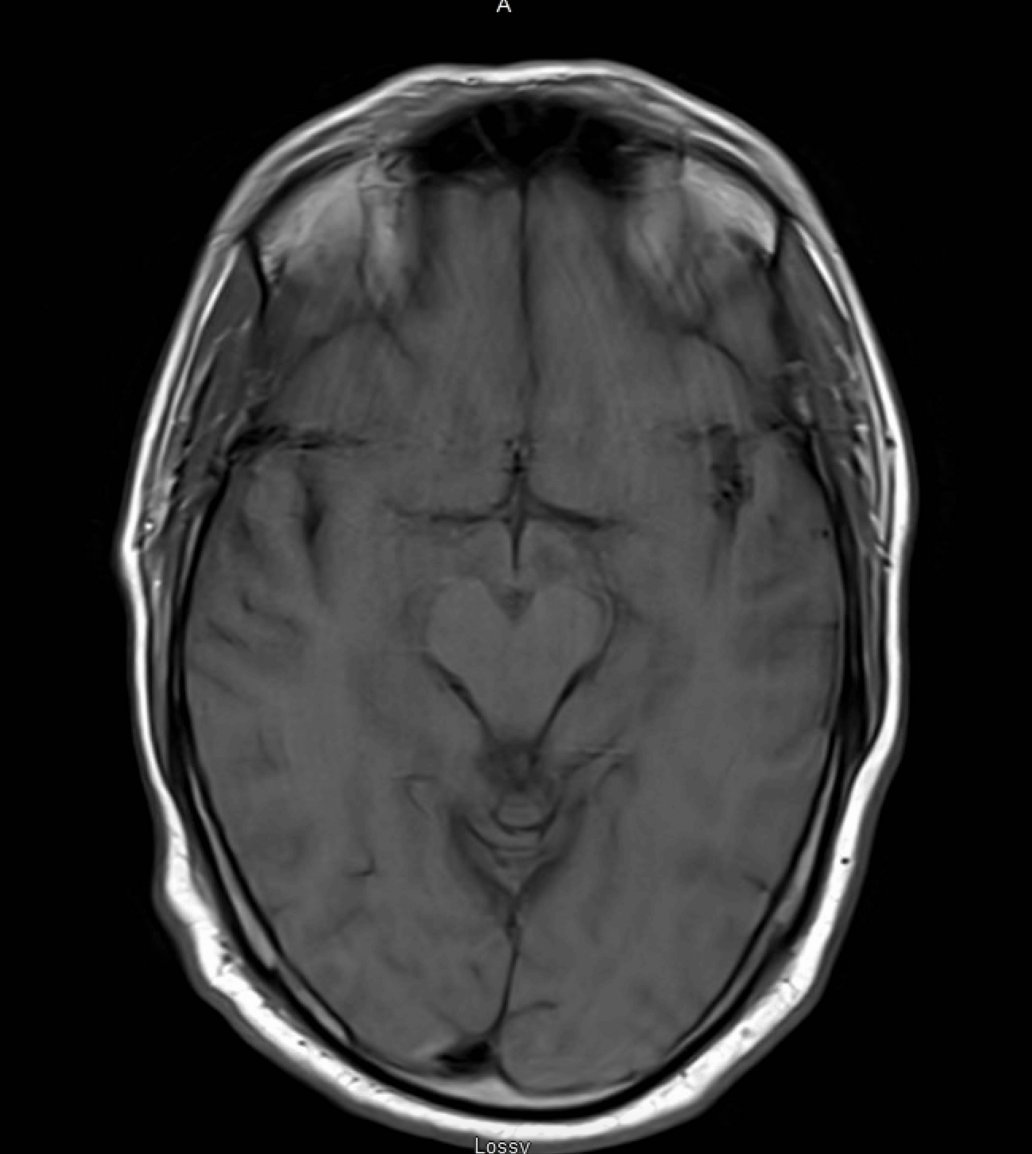
MRI showing no brain involvement

Infectious workup showed positive *Echinococcus* antibody (Table 1). A hepatobiliary scan showed no hepatic involvement (Figure 7). Brain MRI was normal (Figure 8).

**Table 1:** Infectious workup

Test Name	Result	Reference
HIV 1/2 Ag/Ab	Negative	Negative
Mono screen Ab	Negative	Negative
Malaria antigen	Negative	Negative
Giemsa stain	Negative	Negative
T. Cruzi Ab	0.3, Negative	Negative: 1.0 or Less
Toxoplasma Ab	5.1, Negative	Negative <8.0
Amebiasis Ab	Negative	Negative
Taenia solium Ab	Negative	Negative
Echinococcus Ab	Positive	Negative

Based on the imaging findings, positive *Echinococcus* serology, and epidemiological context, a diagnosis of cardiac hydatid cyst (Echinococcus granulosus) was made. Albendazole 400 mg twice daily was initiated. Given the cyst’s size, location, and risk of catastrophic complications (e.g., rupture, embolism, conduction block), surgical excision was performed.

The surgery was successful. The patient had an uncomplicated postoperative course and was discharged on albendazole (400 mg twice daily). At one-month follow-up, he was afebrile, hemodynamically stable (HR 70-80 bpm), and without complaints.

## Discussion

This case illustrates the diagnostic complexity and successful management of a rare cardiac hydatid cyst isolated to the IVS. Cardiac hydatidosis is a rare manifestation, occurring in only 0.02-2% of all hydatid disease cases, with IVS involvement representing a mere 4% of these cardiac cases [4,5,14]. The nonspecific and often prolonged nature of symptoms, such as chest pain, dyspnea, and fatigue, frequently leads to diagnostic delays [2,6].

Our patient’s initial presentation with fever, cough, markedly elevated inflammatory markers (CRP, procalcitonin), and elevated troponin understandably raised suspicion for sepsis or acute myocarditis. The discovery of a high-grade AV block was a critical turning point. Conduction abnormalities are a well-recognized complication of cardiac echinococcosis, particularly with septal involvement, where the cyst can directly compress or inflame the conduction system [5,8,12,15]. This aligns with a reported case where complete heart block was the sole manifestation of a right atrial hydatid cyst; in that instance, the patient declined surgery and was managed with a permanent pacemaker and albendazole therapy [8]. In our case, the junctional escape rhythm indicated significant compromise of the cardiac conduction system, necessitating urgent intervention.

Imaging is the cornerstone of diagnosis. Transthoracic and transesophageal echocardiography typically reveal a well-circumscribed, anechoic, or complex cystic mass, often with a multi-layered wall or internal echoes suggesting daughter cysts [4,5,8]. However, advanced cross-sectional imaging with cardiac MRI and CT is indispensable for precise anatomical characterization, surgical planning, and ruling out extracardiac involvement. MRI is superior for tissue characterization, typically showing a hypointense signal on T1-weighted images and a hyperintense signal on T2-weighted images for the fluid content. It can also identify pathognomonic signs like the "water-lily" sign from detached membranes and demonstrate peripheral capsule enhancement on late gadolinium-enhancement sequences, indicative of the pericystic inflammatory reaction [2,6,14,16]. CT is excellent for demonstrating cyst morphology, fluid density, and the presence of wall calcification, which is suggestive of a chronic or inactive cyst [4,5,14].

Serological testing for *Echinococcus*, while a helpful adjunct, has significant limitations, with reported sensitivities varying widely. False-negative results occur in approximately 20-25% of cases, especially with intact, calcified, or isolated pulmonary or cardiac cysts [2,8,9]. The absence of blood eosinophilia, as seen in our patient and reported in other cases, further diminishes the reliability of these laboratory markers for ruling out the diagnosis [8,9]. Therefore, a negative serology or normal eosinophil count should not preclude the diagnosis when imaging findings are suggestive.

The patient’s exposure history was pivotal in guiding the diagnostic process. His three-year residence in India, a region where cystic echinococcosis is endemic, coupled with his reported contact with stray dogs, the definitive host for *Echinococcus*​​​​*granulosus*, provided strong epidemiological support for the diagnosis [2]. The extended latency period, which can span years to decades between initial infection and clinical manifestation, explains how the cyst could present long after he left the endemic area [2,5].

The management of cardiac hydatid cysts is fundamentally different from that of hepatic cysts. Unlike in abdominal echinococcosis, where a "watch-and-wait" approach may be appropriate for inactive cysts, the presence of a cardiac cyst is generally considered an indication for surgical removal due to the high risk of life-threatening complications such as rupture, embolism, tamponade, or fatal arrhythmias [2,3,6,14]. Surgical principles emphasize the imperative to avoid intraoperative rupture. This is achieved by carefully sterilizing the cyst cavity with protoscolicidal agents (e.g., hypertonic saline, iodine, or silver nitrate) before manipulation, complete evacuation of contents, excision of the cyst wall, and secure closure of the residual cavity [5,15,17].

Adjuvant medical therapy with albendazole is a critical component of management. Preoperative administration aims to sterilize the cyst, thereby reducing the risk of secondary implantation from accidental spillage. Postoperative therapy is recommended to eradicate any residual microscopic disease and prevent recurrence [2,5,15]. Our patient received albendazole perioperatively in accordance with these established guidelines. Given that local recurrence or secondary metastatic disease can manifest many years after initial treatment, long-term clinical and imaging follow-up for a minimum of 10 years is advised [2].

With timely diagnosis and a combination of complete surgical excision and adjunctive antiparasitic therapy, the prognosis is generally favorable. However, mortality rates can be significant, historically reported up to 30%, particularly in cases complicated by cyst rupture [2,18]. Our patient’s uneventful recovery and clinical stability at the one-month follow-up align with the excellent outcomes achievable with proactive, multidisciplinary management.

## Conclusions

This case underscores that cardiac echinococcosis, though rare, must be considered in the differential diagnosis of patients presenting with fever, intracardiac mass, and conduction disturbances, especially those with a history of residence or travel to endemic regions, even in the remote past. Isolated involvement of the interventricular septum is an uncommon presentation. Diagnosis requires a high index of suspicion and relies heavily on multimodal cardiac imaging (echocardiography, CT, and MRI), as serologic tests can be falsely negative. Once diagnosed, management should be urgent and involve a combination of surgical excision and perioperative albendazole therapy to prevent catastrophic complications and ensure a favorable outcome. A meticulous travel and exposure history remains an essential component of the diagnostic evaluation.
